# Tissue-Specific Effects of Vitamin E Supplementation

**DOI:** 10.3390/ijms17071166

**Published:** 2016-07-19

**Authors:** Eugene Jansen, Dale Viezeliene, Piet Beekhof, Eric Gremmer, Leonid Ivanov

**Affiliations:** 1 Centre for Health Protection, National Institute for Public Health and the Environment, PO Box 1, 3720 BA Bilthoven, The Netherlands; piet.beekhof@rivm.nl (P.B.); eric.gremmer@rivm.nl (E.G.); 2 Biochemistry Department, Medical Academy, Lithuanian University of Health Sciences, Eiveniu 4, LT-50009 Kaunas, Lithuania; dale.viezeliene@lsmuni.lt (D.V.); Leonid.Ivanov@lsmuni.lt (L.I.)

**Keywords:** vitamin E, α-tocopherol, oxidative stress, MCP-1, PAI-1, resistin, Il-6, TNF-α

## Abstract

A multivitamin and mineral supplementation study of 6 weeks was conducted with male and female mice. The control group received a standard dose of vitamins and minerals of 1× the Recommended Daily Intake (RDI), whereas a second group received 3× RDI. A third group received a high dose of vitamin E (25× RDI), close to the upper limit of toxicity (UL), but still recommended and considered to be harmless and beneficial. The high dose of vitamin E caused a number of beneficial, but also adverse effects. Different biomarkers of tissue toxicity, oxidative stress related processes and inflammation were determined. These biomarkers did not change in plasma and erythrocytes to a large extent. In the liver of male mice, some beneficial effects were observed by a lower concentration of several biomarkers of inflammation. However, in the kidney of male mice, a number of biomarkers increased substantially with the higher dose of vitamin E, indicating tissue toxicity and an increased level of inflammation. Since this dose of vitamin E, which is lower than the UL, cause some adverse effects, even after a short exposure period, further studies are required to reconsider the UL for vitamin E.

## 1. Introduction

Vitamins are generally considered beneficial for a number of physiological processes, such as anti-oxidant status [1]. Also, the risk for several (chronic) diseases can be reduced by an adequate vitamin status [2,3,4]. Consequently, including an efficient marketing strategy, supplementation of multi-vitamins is very popular in the general population [5]. The anti-oxidant properties were partially attributed to vitamin E, a fat-soluble vitamin, which is a key component in the detoxification of oxidation processes, mainly in rodents [6]. In particular, lipid peroxidation of polyunsaturated fatty acids can be inactivated by the presence of membrane-bound tocopherols [7]. There have recently been a number of human studies, where the intake of high single doses of vitamin E can have detrimental effects [8], such as a higher risk for cardiovascular diseases and prostate cancer [9] and even higher mortality [10,11,12]. Taking supplements with high doses of vitamin E is not recommended, since vitamin E can not only work as an anti-oxidant but apparently also as a pro-oxidant [13].

In this study, we have examined the effects of three-fold RDI of multi-vitamins and minerals in mice. In addition, a high dose of 25-fold RDI of vitamin E was used, which is still below the established level of toxicity. In both exposure studies, possible adverse effects of vitamin E on biomarkers of oxidative stress, redox status and liver and kidney were monitored in the circulation (plasma and erythrocytes) and in liver and kidney tissue.

## 2. Results

### 2.1. Plasma

The effect of exposure to feeds with various vitamin and mineral compositions was studied by measuring a number of plasma biomarkers. Because the plasma volume was limited, only a selected set of biomarkers could be measured. These biomarkers were: the enzymes ALP and ALT, a biomarker of oxidative stress (ROM) measuring the total hydroperoxides, the total antioxidant status (BAP) and the total thiol levels in proteins (TTP).

**Figure 1:**
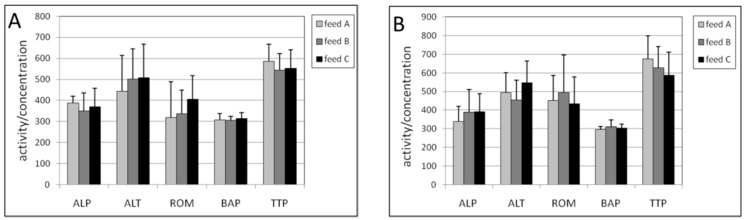
(**A**) Plasma activities in male; and (**B**) female mice of alkaline phosphatase (ALP), alanine aminotransferase (ALT) and plasma concentrations of reactive oxygen metabolites (ROM), biological antioxidant potential (BAP) and total thiol levels in proteins (TTP) after exposure to the three different diets. The levels of ALP, ALT and BAP were multiplied by a factor of 3, 10 and 0.1, respectively to fit in the figure. The levels of feed B were tested statistically relative to those of feed A, and the levels of feed C relative to those of feed B. No statistical differences were found.

In Figure 1 it is shown that exposure to feed B and feed C did not cause a statistically significant effect on these biomarkers, both in male and female mice.

### 2.2. Erythrocytes

**Figure 2:**
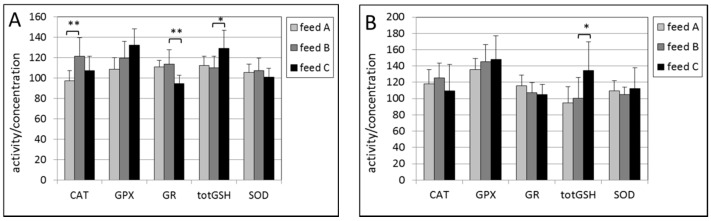
(**A**) Activities of catalase (CAT), glutathion peroxidase (GPX), gluthation reductase (GR), superoxide dismutase (SOD) and concentration of total glutathione (totGSH) in erythrocytes from male (Figure A); and (**B**) female mice. The levels of CAT, GPX, GR and SOD were multiplied by a factor of 10, 5, 0.3 and 2, respectively to fit in the figure. The levels of feed B were tested statistically relative to those of feed A, and the levels of feed C relative to those of feed B. * *p* < 0.05, ** *p* < 0.01.

In the erythrocytes of male mice, the effects of the intake of feed B compared with feed A was a statistically significant increase in activity of CAT (*p* = 0.002) only, as shown in Figure 2A. No effects were observed in erythocytes in female mice (Figure 2B).

High vitamin E content (feed C) caused a statistically lower activity of GR (*p* = 0.003) in male mice and a higher concentration of totGSH (*p* = 0.01) as shown in Figure 2A. In female mice a higher concentration of totGSH was observed with a *p*-value of 0.03 (Figure 2B).

### 2.3. Liver

**Figure 3:**
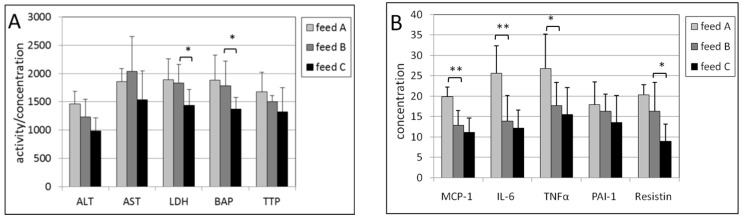
(**A**) Activities of ALT, AST and LDH, and concentrations of BAP and TTP in the post-mitochondrial supernatant of liver tissue of male mice. The levels of LDH, BAP and TTL were multiplied by a factor of 0.2, 10 and 20, respectively to fit in the figure; (**B**) Concentrations of MCP-1, IL-6, TNF-α, PAI-1 and resistin in the post-mitochondrial supernatant of liver tissue of male mice. The levels of IL-6, TNFα and resistin were multiplied by a factor of 4 to fit in the figure. The levels of feed B were tested statistically relative to those of feed A, and the levels of feed C relative to those of feed B. * *p* < 0.05, ** *p* < 0.01.

**Figure 4:**
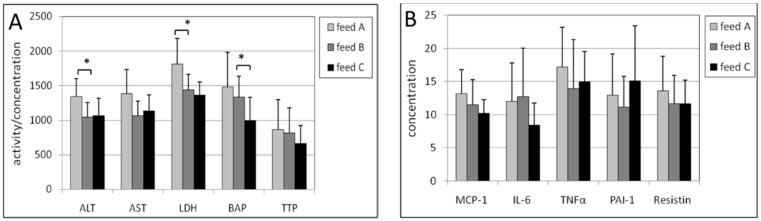
(**A**) Activities of ALT, AST and LDH, and concentrations of BAP and TTP in the post-mitochondrial supernatant of liver tissue of female mice. The levels of AST, LDH, BAP and TTL were multiplied by a factor of 0.5, 0.2, 10 and 10, respectively to fit in the figure; (**B**). Concentrations of MCP-1, IL-6, TNF-α, PAI-1 and resistin in the post-mitochondrial supernatant of liver tissue of female mice. The levels of IL-6, TNF-α and resistin were multiplied by a factor of 4, 3 and 4, respectively to fit in the figure. The levels of feed B were tested statistically relative to those of feed A, and the levels of feed C relative to those of feed B. * *p* < 0.05.

In the liver of male mice, the intake of feed B compared with feed A caused a statistically significantly lower concentration of three inflammation biomarkers, being MCP-1 (*p* = 0.010), IL-6 (*p* = 0.0015) and TNF-a (*p* = 0.017) (Figure 3). The lower levels in ALT, LDH, BAP, PAI-1 and resistin were not statistically significant. In female mice, statistically significantly lower activities of ALT (*p* = 0.014) and LDH (*p* = 0.016) were found (Figure 4). Also, almost all the other biomarkers were decreased but not in a statistically significant manner.

The effects of feed C compared with feed B were observed by a statistically significant decrease in the activity of LDH (*p* = 0.013), BAP (*p* = 0.022), and resistin (*p* = 0.015) for male mice (Figure 3). The decreases in the other biomarkers were not statistically significant (*p*-values for ALT and AST were both 0.07). In female mice only BAP exhibited a lower concentration with a *p*-value of 0.014 (Figure 4).

### 2.4. Kidney

**Figure 5:**
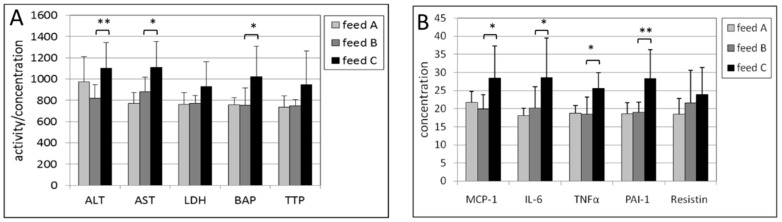
(**A**) Activities of ALT, AST and LDH, and concentrations of BAP and TTP in the post-mitochondrial supernatant of kidney tissue of male mice. The levels of ALT, LDH, BAP and TTL were multiplied by a factor of 10, 0.1, 5 and 20, respectively, to fit in the figure; (**B**) Concentrations of MCP-1, IL-6, TNF-α, PAI-1 and resistin in the post-mitochondrial supernatant of kidney tissue of male mice. The levels of MCP-1, IL-6, TNF-α and resistin were multiplied by a factor of 5, 10, 20 and 0.25, respectively, to fit in the figure. The levels of feed B were tested statistically relative to those of feed A, and the levels of feed C relative to those of feed B. * *p* < 0.05, ** *p* < 0.01.

**Figure 6:**
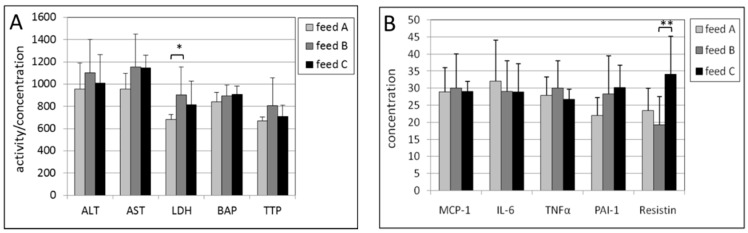
(**A**) Activities of ALT, AST and, LDH, and concentrations of BAP and TTP in the post-mitochondrial supernatant of kidney tissue of female mice. The levels of ALT, LDH, BAP and TTL were multiplied by a factor of 10, 0.1, 5 and 20, respectively, to fit in the figure; (**B**) Concentrations of MCP-1, IL-6, TNF-α, PAI-1 and resistin in the post-mitochondrial supernatant of kidney tissue of female mice. The levels of MCP-1, IL-6, TNF-α and resistin were multiplied by a factor of 5, 10, 20 and 5, respectively, to fit in the figure. The levels of feed B were tested statistically relative to those of feed A, and the levels of feed C relative to those of feed B. * *p* < 0.05; ** *p* < 0.01.

In the kidney, no effects of the intake of feed B compared with feed A were found in male mice (Figure 5A,B). For female mice, only a statistically significant higher activity of LDH was observed with a p-value of 0.014 (Figure 6).

Significant effects of high vitamin E intake (feed C) compared with feed B were found for all biomarkers in male mice. Statistically significant increases were found for ALT (*p* = 0.005), AST (*p* = 0.020), BAP (*p* = 0.028), MCP-1 (*p* = 0.014), IL-6 (*p* = 0.047), TNF-α (*p* = 0.031) and PAI-1 (*p* = 0.003) (Figure 5A,B). The higher concentration of TTP was not statistically significant (*p* = 0.11). In female mice, only resistin showed a statistically significantly higher concentration with a *p*-value of 0.007 (Figure 6B).

In order to test whether there were interactions between the two sex groups, a Bonferroni post-test was performed. There were statistical differences in a number of biomarkers between male and female animals. This was especially observed in a number of biomarkers in groups receiving feed A: in the erythrocytes for GPX, in the liver for MCP-1, IL-6, TNF-a, resistin, AST, BAP and in the kidney for MCP-1, IL-6, TNF-a, resistin. These differences were not observed between the groups which received feed B and feed C.

## 3. Discussion

The current study examined possible effects of increased intake of vitamins and minerals. Three different feeds were given to 6-months-old male and female mice for 6 weeks. In addition to a normal control diet containing 1× RDI of vitamins and minerals, a diet with 3× RDI of vitamins and minerals was given to test whether a moderate increased dose of vitamins and minerals has any effects on biomarkers of tissue damage and inflammation in plasma, erythrocytes, liver or kidney. Many preparations with 3× RDI level of vitamins and minerals can be purchased in drugstores and on the Internet. As a third feed, the second feed (3× RDI) with on top a high dose of vitamin E was given. This high dose is close to the UL for men as established for Europe by EFSA [14] and is also available in drugstores as a single food supplement for human use.

The feed with a three-fold dose of vitamins and minerals did not show a statistically significant effect on plasma biomarkers of tissue toxicity or oxidative stress or redox status both in male and female mice. Also in the erythrocyte antioxidant system almost no effects were observed, except for a higher activity of CAT for the male mice.

In the liver of male mice, a number of non-significant observations were made such as a decrease in enzymes of tissue damage and some inflammation biomarkers. The lower concentrations of these biomarkers of inflammation can be a beneficial effect of the higher dose of vitamins on the liver. A similar effect is shown in female mice, although the effect is smaller and only statistically significant for certain liver enzymes, but not for the inflammation biomarkers.

In the kidney of male and female mice, almost no effect could be observed (only for resistin) as a result of the three-fold dose of multi-vitamin and minerals.

The high dose vitamin E showed no statistically significant effects on plasma biomarkers, although there are some non-significant trends such as higher activities and concentrations of ALT and ROM in male mice and a lower concentration of TTP in female mice, which may indicate a slightly increased oxidative stress situation. Both ROM as an indicator of oxidative stress and TTP as a biomarker of redox status have proven its value in large scale studies in several European cohorts [15,16,17,18]. In the erythrocytes a statistical increase in totGSH was observed indicating an improvement in the redox status.

In the liver and kidney, the opposite effects were observed with a higher dose of vitamin E. In the liver, lower concentrations of several biomarkers of tissue toxicity and inflammation were measured, although some effects were not statistically significant. In the kidney, however, substantial higher concentrations in most of the tissue and inflammation biomarkers were observed for the high dose of vitamin E, but only in male mice.

The results of the male and female mice were not pooled together because of an expected different behavior in both sexes on supplementation of vitamins and minerals [19]. It seems that male mice are more susceptible to the effects of higher doses of vitamins and minerals. These gender differences cannot be explained by the current data.

EFSA considered the study by Meydani et al. (1998) [20] as the best scientific evidence for a tolerable upper intake level (UL) for vitamin E. EFSA established an UL for vitamin E of 270 mg/day for adults that was rounded to 300 mg/day. The UL probably will not give rise to adverse health effects for 95% of the people in the general population [14].

In the present study, the application of an oral dose of 25× RDI of vitamin E in mice showed a number of adverse effects on inflammation parameters. Biomarkers of oxidative stress, anti-oxidant capacity and redox status were not affected, as could be possibly expected from human studies [21]. The beneficial effects of normal doses of vitamin E (in the range of the RDI) on these parameters, often observed as a lower inflammation status, is quite contrary when higher dose levels are used as shown in the present study.

Further studies are needed to see whether a new evaluation of the UL should be considered. It should be noted, however, that these results in mice may not be relevant to humans. A recent study with obese children showed a decrease in oxidative stress biomarkers and no effect on inflammation upon supplementation of a high dose of vitamin E, C and selenium [22]. Further studies are required to elucidate the potential beneficial or adverse effects of vitamin E.

## 4. Materials and Methods

### 4.1. Experimental Animals and Exposure Protocol

Balb/c mice (age 6 months) were fed for 6 weeks with three different feeds. Feed A contained 1× RDI of all vitamins and minerals. Feed B contained 3× RDI of all vitamins and minerals, with the exception of fluorine, sodium, chlorine, silicon, nickel, boron, lithium and vanadium. Feed C was comparable with Feed B with the exception of vitamin E which was added at a level of 25× RDI. The exact composition of the three different feeds is shown in Table 1. The feeds were prepared by Research Diet Services BV, Wijk bij Duurstede, The Netherlands.

Each group of mice consisted of ten animals for each dose and gender. All sacks with feed were stored at −20 °C during the experiment. Only a small part of feed that was used in 3 days was stored at +4 °C. The mice were placed in cages (1 mouse/cage) at room temperature (20 °C). One day before termination the mice did not get feed. The organs (liver and kidney) were removed, rapidly cooled on ice until further preparation of extracts.

All procedures were performed according to the Republic of Lithuania Law on the Care, Keeping and Use of Animals (License by the State Veterinary Service for working with laboratory animals No 0200).

### 4.2. Preparation of Blood Samples

Whole blood was collected after mouse decapitation in tubes containing heparin and centrifuged at 4000 rpm for 10 min (1600× *g*) in a Savant centrifuge. The plasma layer and the erythrocytes were frozen immediately and stored in tubes at −80 °C.

All samples were shipped from Kaunas to Bilthoven on dry ice. The samples were received in frozen condition and stored at −80 °C until analysis.

### 4.3. Biochemical Analysis in Blood Samples

Alkaline phosphatase (ALP) and alanine aminotransferase (ALT), both expressed in IU/L, reactive oxygen metabolites (ROM), expressed in U/L, biological antioxidant potential (BAP), expressed in mEq/L, and total thiols (TTP), expressed in µmol/L, were determined in heparin plasma with an auto-analyzer (LX-20 Pro, Beckman Coulter, Woerden, The Netherlands). ALP and ALT were determined using kits from Beckman-Coulter. ROM and BAP were determined using kits from Diacron, Grosseto, Italy and TTP was determined with a kit from RelAssay, Gaziantep, Turkey. These three assays have been adapted for use on the LX20 auto-analyzer. Intra-assay coefficients of variation were 3.9% for ALP, 1.4% for ALT, 1.2% for LDH, 2.1% for ROM, 1.7% for BAP and 0.9% for TTP. Both short- and long-term stability of these biomarkers in the processing and storage are very good [23,24,25].

Catalase (CAT), expressed in µmol/mmol Hb, glutathion peroxidase (GPX), expressed in kU/mmol Hb, gluthation reductase (GR), expressed in IU/mmol Hb, total glutathione (totGSH), expressed in µmol/mmol Hb, superoxide dismutase (SOD), expressed in mU/mmol Hb, and hemoglobin were measured in erythrocytes with an auto-analyzer (LX-20 Pro, Beckman Coulter). Kits for GPX, GR and SOD were obtained from Randox, Crumlin, UK. CAT and totGSH were measured with in-house developed assays. Hemoglobin was measured with a kit from Beckman-Coulter. Intra-assay coefficients of variation were 2.3% for CAT, 1.3% for GPX, 1.8% for GR, 4.2% for totGSH and 1.2% for SOD.

### 4.4. Preparation of Tissue Samples

Liver and kidney were homogenized in 3 volumes of homogenization buffer (50 mmol/L Tris-HCl at pH 7.6, 5 mmol/L MgCl_2_, 60 mmol/L KCl and 25 mmol/L sucrose). The tissue homogenates were centrifuged at 3000 rpm for 10 min (1000× *g*) in a Beckman J2-21 centrifuge. The supernatant was centrifuged again at 10,000 rpm for 15 min (12,000× *g*) in the same centrifuge. The second post-mitochondrial supernatant (PMS) was immediately frozen at −80 °C.

### 4.5. Biochemical Analysis in Tissue Samples

ALT, aspartate aminotransferase (AST), lactate dehydrogenase (LDH) all expressed in IU/g protein, BAP expressed in mEq/g protein, TTP, expressed in µmol/g protein) and total proteins were determined in liver and kidney tissues with an auto-analyzer (LX-20 Pro, Beckman-Coulter). Intra-assay coefficients of variation were 0.8% for ALT, 1.1% for AST, 3.3% for LDH, 2.0% for BAP, 1.8% for TTP and 2.6% for total protein.

Monocyte chemoattractant protein-1 (MCP-1), interleukin-6 (IL-6), tumor necrosis factor alpha (TNF-α), plasminogen activator inhibitor-1 (PAI-1), and resistin, all expressed in pg/g protein, were determined with a Mouse Adipokine kit MADKMAG-71K from Millipore, Amsterdam, The Netherlands using the Luminex technique. The measurements were performed with the Luminex100 from Luminexcorp, ‘s Hertogenbosch, The Netherlands. Intra-assay coefficients of variation were 10.0% for MCP-1, 6.4% for IL-6, 4.9% for TNF-α, 7.4% for PAI-1 and 5.2% for resistin.

### 4.6. Statistical Analysis

The statistical analysis was carried out between groups that received feed A (1× RDI) and feed B (3× RDI) and between the groups that consumed feed B (3× RDI) and feed C (3× RDI + Vitamin E). The statistical analysis was not carried out between groups that received feed A and feed C, because two variables were changed in these feeds, being the amount of the RDI and additional vitamin E.

In a first approach, a two-way analysis of variance (ANOVA) was carried out. From these analyses the statistical significances were calculated. The results were expressed as the mean ± standard error of mean. Statistical significances were set at *p* < 0.05 or *p* < 0.01.

A second approach was used to determine possible cross-correlations between the different sex groups. This was done by a univariate ANOVA, followed by a post-analysis using the Bonferroni correction to adjust for multiple comparisons. The ANOVAs were performed using the Excel program. The Bonferroni test was performed using Graphpad Prism (www.graphpad.com).

## Figures and Tables

**Table 1:** Composition of the different feeds with respect to vitamins and minerals.

Compound	Feed A	Feed B	Feed C
	mg/kg Diet	mg/kg Diet	mg/kg Diet
Vitamin A	0.72	2.16	2.16
Vitamin B1	5	15	15
Vitamin B2	7	21	21
Vitamin B3	15	45	45
Vitamin B5	16	48	48
Vitamin B6	6	18	18
Biotin	0.2	0.6	0.6
Folic acid	0.5	1.5	1.5
Vitamin B12	0.01	0.03	0.03
Vitamin C	50	150	150
Vitamin D3	0.025	0.075	0.075
Vitamin E	22	66	550
Vitamin K	0.3	0.9	0.9
Choline	1250	3750	3750
Calcium	5000	15,000	15,000
Potassium	3000	9000	9000
Phosphorus	2000	6000	6000
Iron	35	105	105
Magnesium	500	1500	1500
Copper	6	18	18
Selenium	0.15	0.45	0.45
Zinc	10	30	30
Chromium	1	3	3
Iodine	0.15	0.45	0.45
Manganese	10	30	30
Molybdenum	0.15	0.45	0.45
Fluorine	1	1	1
Sodium	500	500	500
Chlorine	500	500	500
Silicon	5	5	5
Nickel	0.5	0.5	0.5
Boron	0.5	0.5	0.5
Lithium	0.1	0.1	0.1
Vanadium	0.1	0.1	0.1

## Abbreviations

BAP	(biological antioxidant potential)
IL-6	(interleukin-6)
MCP-1	(monocyte chemoattractant protein-1)
tPAI-1	(total plasminogen activator inhibitor)
PMS	(post-mitochondrial supernatant)
RDI	(recommended daily intake)
ROM	(reactive oxygen metabolites)
TNF-alpha	(tumor necrosis factor-alpha)
TTL	(total thiol levels)
